# Which design to evaluate complex interventions? Toward a methodological framework through a systematic review

**DOI:** 10.1186/s12874-019-0736-6

**Published:** 2019-05-07

**Authors:** Laetitia Minary, Justine Trompette, Joëlle Kivits, Linda Cambon, Cyril Tarquinio, François Alla

**Affiliations:** 1 University of Lorraine, EA 4360 APEMAC, Nancy, France; 2 Ireps Grand-Est, Nancy, France; 3 University of Bordeaux, INSERM, Bordeaux Population Health Research Center, Bordeaux, France

**Keywords:** Research methods, Study design, Public health, Health behaviour

## Abstract

**Background:**

Evaluation of complex interventions (CI) is challenging for health researchers and requires innovative approaches. The objective of this work is to present the main methods used to evaluate CI.

**Methods:**

A systematic review of the scientific literature was conducted to identify methods used for the evaluation of CI. We searched MEDLINE via PubMed databases for articles including an evaluation or a pilot study of a complex intervention, published in a ten-year period. Key-words of this research were (“complex intervention*” AND “evaluation”).

**Results:**

Among 445 identified articles, 100 research results or protocols were included. Among them, 5 presented 2 different types of design in the same publication, thus our work included 105 designs. Individual randomized controlled trials (IRCT) represented 21.9% (*n* = 23) of evaluation designs, randomized clinical trials adaptations 44.8% (*n* = 47), quasi -experimental designs and cohort study 19.0% (*n* = 20), realist evaluation 6.7% (*n* = 7) and other cases studies and other approaches 8.6% (*n* = 9). A process/mechanisms analysis was included in 80% (*n* = 84) of these designs.

**Conclusion:**

A range of methods can be used successively or combined at various steps of the evaluation approach. A framework is proposed to situate each of the designs with respect to evaluation questions. The growing interest of researchers in alternative methods and the development of their use must be accompanied by conceptual and methodological research in order to more clearly define their principles of use.

## Background

Much has been written about complex interventions (CIs) in health since they were defined by the Medical research council (MRC) [9]. These interventions cover fields as diverse as health services, health behavior change interventions, and health promotion and social policy interventions. Such current interest in CIs reflects the challenge they represent for research communities, practitioners and decision-makers [13]. Indeed CIs are context-dependent, which raises the question of their transferability [6]. When health interventions are considered to be complex, open and varying approaches to their evaluation are required. Individual randomized clinical trials (IRCT), guaranteeing a causal inference between interventions and effects and consequently representing the gold standard to evaluate their efficacy, are not always relevant (e.g. for the evaluation of a health policy) or sufficient in this field. Firstly, the complexity of interventions is difficult to reduce to fit the experimental framework and cannot be strictly standardized [12]. Secondly, IRCTs are known to be limited when the implementation context is a determinant of the result. Indeed experimental conditions differ from those of real life in many significant way (e.g. volunteer and trained professionals, standardised procedures, specific context). The results may therefore not be transferable [7, 42] to a non-experimental context [37]. Moreover, because of the interaction between interventions and their environments, some biases encountered in clinical research (such as sampling bias) could be reinforced [37]. For example, the effect of an intervention can vary across individuals [44]; the same dose may have less effect if there is less need for it [41]. Finally, beyond the efficacy of the intervention, practitioners and policymakers also need data about the conditions of implementation and the mechanisms of the intervention in order to generalize effective interventions and to adapt them into their specific context [6, 32, 34, 39]. Evaluation models that attempt to overcome the limitations of IRCTs have been explored for a long time, especially in the fields of social sciences and educational sciences [8], such as RCT adaptations (e.g. pragmatic RCT [39], cluster RCT [28]) or alternative designs (e.g. realist evaluation [31]). Alternative and adaptive models/frameworks provide a better understanding of the mechanisms of the intervention and can identify contextual aspects of interventions likely to influence the results. In 2015, the MRC provided guidance for process evaluation [26], which constitutes major progress as this guidance addresses the challenge of considering intervention processes and mechanisms as part of the whole evaluation approach. Interest of researchers in these methods has grown over recent years. The challenge is now to identify the design fitting with the object of evaluation. The MRC guidance must be operationalized, especially to better integrate theories [5, 27]. Moreover, alternative methodological approaches are the subject of conceptual and methodological debates [3]. The actual methodological gold standards led to an under-use of alternatives methodological approaches. Defining principles of use of evaluation methods could help researchers to identify the best method according to their research question. The objective of the current study is to present the main methods used for evaluating CI’s and to propose a framework allowing the classification of these methods according to the evaluation questions.

## Methods

A systematic review of the scientific literature was conducted to identify methods used for the evaluation of CI following the Preferred Reporting Items for Systematic Reviews and Meta-Analyses (PRISMA) guidelines [24]. The research strategy has been designed to identify articles written by authors who evaluate complex interventions in the field of health (clinical care and health services research; health promotion and prevention).

### Search strategy

A research on MEDLINE via Pubmed database for articles published in a ten-years period (January 2004 to December 2014) was undertake with the following key words: (« complex intervention[s] » AND « evaluation ») in title and/or abstract and/or body text. The start date was chosen to identify studies that could integrate the first MRC guidance [9].

### Selection of the articles

Articles retrieved with search strategy were included according to: 1. language – written in English or French – 2. type of article - research articles and protocols of any design were included. Conceptual framework, reviews/meta-analysis, feasibility and/or pilot studies, methodology articles, abstracts, chapter of book, comment/letter, congress and oral presentation were excluded – 3. subject of the article – an evaluation or pilot study of a complex intervention (as defined by authors).

Two independently working reviewers carried out initial title and abstract screening (to exclude papers that were definitely ineligible) followed by a detailed full-text screening of remaining papers (to exclude papers not meeting all inclusion criteria, with reasons for exclusion recorded). Any disagreements between reviewers were resolved with a third reviewer.

### Analysis

A content analysis of full texts of selected articles was undertaken in order to identify methods of evaluation used by researchers. We constructed a data collection grid allowing the extraction of the following elements: author, date, type of article (protocol study or original research), investigated field (health promotion/prevention, clinical care/health services research), evaluation (yes/no), pilot study (yes/no), type of design (individual RCT, pragmatic RCT, cluster RCT, pragmatic and cluster RCT, quasi-experimental design, cohort study, realist evaluation, other case studies, others), process evaluation (yes/no), quantitative/qualitative/mixed approach..

## Results

**Fig. 1:**
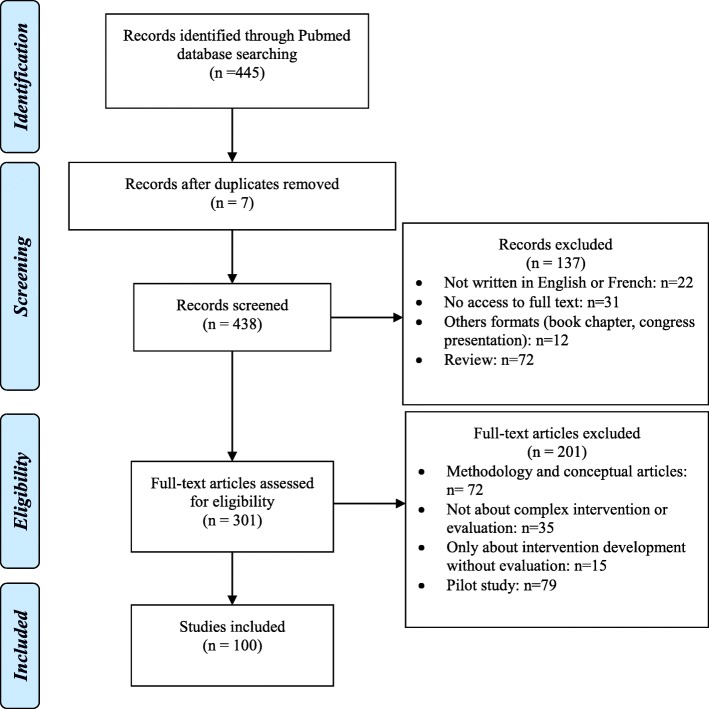
PRISMA flow chart

The search identified 445 potential articles: 338 were excluded (Fig. 1). They were distributed as follow: 7 were duplicated, 72 review or meta-analysis, 79 pilots studies, 72 methodology articles and 12 from other types (e.g. letter/comment); 22 were not written in English or French; 15 focused only on intervention development without evaluation; 35 were not about complex interventions or their evaluation and 31 were not accessible (journals not available). We kept the articles combining pilot study and evaluation (*n* = 11).

The 100 remaining papers covered research results (*n* = 52) or research protocols (*n* = 48) whose thematic were clinical care/health services research (*n* = 72) or health promotion/prevention (*n* = 28).

Among the 100 articles retained in the analysis, 5 presented 2 different types of design in the same publication, thus our work included 105 designs identified.

Individual RCT represented 21.9% (*n* = 23) of evaluation designs (Table 1).

**Table 1:** Distribution of evaluation designs (*n* = 105)

	Total *N* (%)	Process and mechanism evaluations *N* (%)	
Individual randomized trials	23 (21.9)	14 (13.3)	
Randomized trial adaptations	47 (44.8)	43 (40.9)	
*Cluster randomized trials*	*25 (23.8)*	*23 (21.9)*	
*Pragmatic trials*	*9 (8.6)*	*8 (7.6)*	
*Cluster and pragmatic*	*13 (12.4)*	*12 (11.4)*	
Alternative methods to RCT	36 (34.3)	27 (25.7)	
*Quasi-experimental*	*14 (13.3)*	*12 (11.4)*	
*Cohort study*	*6 (5.7)*	*1 (0.1)*	
*Realist evaluation*	*7 (6.7)*	*7 (6.7)*	
*Case studies and other approaches*^*a*^	*9 (8.6)*	*7 (6.7)*	

^a^(Triangulated approach, goal-based evaluation, constructivist evaluation illuminative evaluation)

RCTs adaptations represented 44.8% (*n* = 47) of evaluation designs, including pragmatic RCTs 8.6% (*n* = 9), cluster RCTs 23.8% (*n* = 25) and both pragmatic and cluster RCTs 12.4% (*n* = 13).

Alternative methods to RCT represented 34.3% (*n* = 36) of evaluation designs, including quasi -experimental designs and cohort study 19.0% (*n* = 20), realist evaluation 6.7% (*n* = 7) and other cases studies and other approaches 8.6% (*n* = 9).

A process/mechanisms analysis was used for 80.0% (*n* = 84) of these articles (60.9% (*n* = 14) within individual RCT and 84.3% (*n* = 70) with other adapted or alternative designs) (Table 1).

The methods were used both in health promotion/prevention (*n* = 29) and clinical care/health services research fields (*n* = 60) (Table 2). However, we observed that process evaluation tended to be more used in health promotion/prevention field.

**Table 2:** Distribution of evaluation designs according to the evaluation field (*n* = 105)

	Health promotion/Prevention (*n* = 29)		Clinical care/ Health services research (*n* = 76)	
	Total *n* (% ^a^)	Including process and/or mechanism evaluation *n* (%^b^)	Total *n* (%^a^)	Including process and/or mechanism evaluation n (%^b^)
Individual randomized trials	6 (20.7)	5 (83.3)	17 (22.4)	9 (52.9)
Randomized trial adaptations	9 (31.0)	9 (100)	37 (48.7)	34 (91.9)
*Cluster randomized trials*	*5 (17.2)*	*5 (100)*	*19 (25)*	*18 (94.7)*
*Pragmatic trials*	*1 (3.4)*	*1 (100)*	*8 (10.5)*	*7 (87.5)*
*Cluster and pragmatic*	*3 (10.3)*	*3 (100)*	*10 (13.2)*	*9 (90)*
Alternative methods to RCT	14 (48.3)	13 (92.9)	22 (28.9)	14 (63.6)
*Quasi-experimental*	*7 (24.1)*	*6 (85.7)*	*7 (9.2)*	*6 (85.7)*
*Cohort study*	*0 (0)*	*0*	*6 (7.9)*	*1 (16.7)*
*Realist evaluation*	*2 (6.9)*	*2 (100)*	*5 (6.6)*	*5 (100)*
*Case studies and other approaches*	*5 (17.2)*	*5 (100)*	*4 (5.3)*	*2 (50)*

^a^n/ number of design (*N* = 108)

^b^n/number of such type of desing (for example: 87.5% of Individual RCT are combined with process and/or mechanism evaluation)

## Discussion

This review has identified and analyzed the main methods used for the evaluation of CI. Health promotion/prevention field tends to turned to alternative methods to individual RCT more than clinical field, probably because such field is less imprinted by the biomedical paradigm and is influenced by social science methodologies.

Our research let us to define three main types of methods -non-mutually exclusive because we observed that according to the objective of the researcher, multiple designs may be use during an evaluation. After describing the main used methods, we will analyze how they can be articulated in a global approach.

### Description of main used methods

In order to describe and analyze the main methods other than IRCTs used for the evaluation of CIs, we chose to classify them here into three types: 1) IRCT adaptations; 2) process/mechanisms analysis within trials using mixed approaches; 3) alternative methods to RCT.

1. IRCT adaptations

Some adaptations to RCTs take into account the specific constraints related to the nature of complex interventions allowing them to more closely correspond to real-life conditions. These designs aim to test the effectiveness of interventions in routine clinical practice [35, 36] and therefore maximize their applicability and transferability [30, 35].

#### Pragmatic RCT

Pragmatic trials have the “purpose of informing real world decision about which among the alternative treatments to choose” [39]. One intervention is evaluated against other interventions in routine practice settings [30]. This permits to consider rather than a binary distinction between “explanatory” trials and “pragmatic” trials, there is a pragmatic-explanatory continuum [38]. Such a trial has real pertinence for the evaluation of CIs in that it strengthens the external validity [30]. It may also be adapted to guide the analysis of the feasibility of complex interventions in advance of their implementation, as it assists in the systematic and comprehensive mapping of the human, organisational and resource changes that an intervention will require [39]. However, pragmatic trial is expensive, difficult to implement, and is subject to methodological limitations [10, 28, 30]: one limitation is that the increase of a trial’s “within-study” heterogeneity (eg, variability of practitioners, patient and health care delivery) does not always involve the increase of the external validity by lowering the “between-study” heterogeneity among different trials. Furthermore, in the case where the intervention is designed in a specific combination of practitioners/beneficiary, such trials could led to a dilution of effect in extended populations. Finally, whereas a pragmatic trial can inform on the overall performance of an intervention, it remains very difficult to identify the specific components that explain this effectiveness.

#### Cluster randomized trials

Cluster randomized trials (CRTs) are defined as experiments in which entire social units or clusters of individuals rather than independent individuals are randomly allocated to intervention groups [28]. They are used for interventions that have to be delivered at a group level or where “contamination” between intervention groups must be avoided ([15].). Several variants exist [19, 21]. The advantages of CRTs make them a useful tool for public health research, complex by nature. Indeed, they allow considering a component of this complexity which is the interaction between individuals. For example, in a study which aims to evaluate the effectiveness of smoking cessation program among adolescents [23], the use of such cluster design allowed to identify a group effect concerning tobacco cessation. However, these trials have a high risk of selection and dilution bias, as not all subjects in a cluster participate in the intervention [18]. Other methodological limitations, such as the cluster effect or an imbalance of individual characteristics between clusters, are well known [18, 21]. Furthermore, as a blind design is generally not possible, individual preferences for one or other of the interventions compared can influence the results of the evaluation. Finally, the possibilities and modalities of obtaining consent raise an ethical issue, particularly when exposure to the intervention is difficult to avoid, even in the case of refusal [18, 45].

2) process/mechanisms analysis within trial

As a complement to efficacy analysis, these approaches focus on operative mechanisms: they aim to understand why a specific result was obtained and what could have contributed to it. Indeed, as complex interventions are context dependent, an evaluation of efficacy that does not explain how an intervention produces an effect within a specific context, could led to non-reproducible results. In this context, the Medical Research Council recently published recommendations to guide researchers in their process evaluation approach [25]. Such approach includes analysis of process, components and mechanisms taking into account context. It generally involves the use of qualitative or mixed research methods.

#### Process analysis – process evaluation

Process evaluation within RCTs integrates, within the experimental design, an evaluation of the process in order to understand explanatory elements (the “black box” of a complex intervention) that may influence the outcome [29]. Process evaluation within trials “may aim to examine the views of participants on the intervention; study how the intervention is implemented; distinguish between components of the intervention; investigate contextual factors that affect an intervention; monitor dose to assess the reach of the intervention; and study the way effects vary in subgroups” [29]. Thus attention is paid to parameters that cannot be standardized or controlled within complex intervention evaluation, such as individual perceptions. Oakley et al. also indicated the benefit of process evaluation in discerning whether an intervention may be “inherently faulty (failure of intervention concept or theory)” or “badly delivered” (implementation failure). The advantage of this method is that it does not exclude RCTs, but rather allows for a combination of qualitative and quantitative methods in order to help in the interpretation of the outcome result. Indeed, qualitative methods such as case studies, focus group, interviews or observations help to capture emerging changes in implementation, experiences of the intervention and unanticipated or complex causal pathways in order to to explain quantitative findings. They also help to generate new theory [25]. Conversely, quantitative data would allow to test hypotheses generated by qualitative data [25]. It represents a transfer tool of research results to practice by simultaneously facilitating understanding of the mechanisms (i.e. underlying entities, processes, or structures which operate in particular contexts to generate outcomes of interest - Astbury, 2010) and data reporting by researchers. However, when it is associated with a RCT, the process investigated will probably not be representative of the process observed in real life conditions [29]. Furthermore, according to the objective of the study, the scope of process evaluation is varying. Initially, most of process evaluations were focusing on implementation process without theoretical hypothesis, specifically when there were combined with clinical care individual RCT. Last decade has seen the emergence of theory driven RCT that use theory of change (ToC) as a pragmatic framework which describes how the intervention affects change [14]. Theory informs about how and why an intervention works. It allows a deeper exploration of the interaction between intervention and context through multiple causal pathways, levels of interventions and feedback loops which better reflect the reality of how complex interventions achieve their impact [14]. In allowing for a detailed understanding of how and whether an intervention works and which components of a complex intervention are the most important in achieving impact, they help to reach an even better external validity [14, 43].

#### Realist RCTs

Bonnel et al. [2] have proposed a model integrating exploration of the mechanisms of the intervention through theorization in a “realist approach” combined to RCT. Starting from a criticism by realist evaluators [31] that RCTs fail to understand mechanisms, Bonnel et al. proposed maintaining the realist posture while recognizing the contribution of RCTs. Realist randomized controlled trials are developed as a model balancing experimental design with the necessity of theorising and empirically evaluating how intervention mechanisms interact with context to generate outcomes. They allow evaluations to be focused on refining generalisable intervention theory, as well as accrediting particular interventions as effective or not, as both questions can be examined within modified RCT designs. Thus they constitute a valuable methodology in asking a range of questions about implementation, context, mechanisms, outcomes and normalisation [25] However, realist RCTs are the subject of debate. The major counter-argument evoked by some realist evaluators is that realist RCT do not take into account important elements of complexity of intervention, particularly the characteristics of social interventions (non-linearity, local adaption, feedback loops, emergence, path dependence, and the role of human agency) [22]. Another argument is the difference in the treatment of causation between post-positivist and realist logic due to a different understanding of “mechanism” and to the reliance on correlation between variables as the main analytical strategy [22, 40].

3) alternative methods to RCTs

#### Realist evaluation

The realist perspective has found a welcoming audience among CI evaluators. The idea is to explore mechanisms that are activated by the intervention to produce its effects in a given context. Realist evaluation can provide an explanation on how an intervention functions and in what circumstances [31]. It is based on the “Context-Mechanisms-Effects” principle: the effect of an intervention is the result of the interaction between the supposed mechanism and the context [31]. It implies analyzing not only the intervention results but also its levers. It is based on an iterative procedure whereby successive case studies are conducted. The advantage of this approach in the context of complex intervention evaluation is that it takes into account the mechanisms underlying the intervention and its context of implementation, which provides practitioners and decision-makers with elements of choice. It can also be used when it is impossible to conduct a comparison with a control group by considering all things (other than the intervention) to be otherwise equal. However, this method involves a time-consuming and complicated approach. Moreover, as hypotheses are related to the context, they cannot always be generalized.

#### Natural experiments

The growing interest in comparative effectiveness research, has led to a new interest in quasi-experimental and non-experimental studies due to their greater external validity. Quasi-experimental designs are well known and have several variants [17, 20]. The concept of natural experiments provide an opportunity to evaluate the effects and the process of interventions in real-world settings [20]. Natural experiments usually take the form of an observational study in which the researcher cannot control or withhold the allocation of an intervention to particular areas or communities, but where natural or predetermined variation in allocation occurs [33]. In a context of complex intervention evaluation, it permits to evaluate real world practice, and to have high external validity. It has a particularly strong interest when “there is a reasonable expectation that the intervention will have a significant health impact, but scientific uncertainty remains about the size or nature of the effects; an RCT would be impractical or unethical; and the intervention or the principles behind it have the potential for replication, scalability or generalisability”[11].

However, such design has limitations. The selective exposure to the intervention may create a bias which reduce the capacity of research to conclude on a clear causal inference between intervention and effect [1]. Internal validity would be enhanced in reducing reliance on tenuous assumptions about residual confounding [4].

### Situating designs according to evaluation questions

While some methods predominantly consider the effect of the intervention, other would help to examine implementation, mechanisms of impact, and interactions with contextual factors. RCT adaptations (i.e. pragmatic trials, clusters RCTs) make possible to evaluate intervention effectiveness in conditions closer to real life and thereby to maximize their transferability [6]. Process evaluation trials and realist RCTs contribute to the understanding of interventions mechanisms. In the same way, context by treatment interactions analysis within cluster RCTs, aim at improving theorization about the relationship between social phenomena and health [16]. Process evaluation is also used within natural experiment or quasi-experimental studies. These last designs are particularly important to consider when the aim is to produce data on interventions conducted in real-life conditions or when a RCT cannot be performed (e.g. evaluation of a health policy).

Our review method does not guarantee the comprehensiveness. Especially the selection with the key words “complex intervention*” and “evaluation” does not allow to be exhaustive for the interventions which could be defined as complex but which are not qualified as such by their authors. In the same way, the Pubmed database references publications in the field of health but underestimates those published in other disciplines, such as education sciences and social sciences.

However, our objective was not to be exhaustive but to identify what was the range of methods used by researchers who identify themselves as researchers in this new field of “complex intervention evaluation”.

**Fig. 2:**
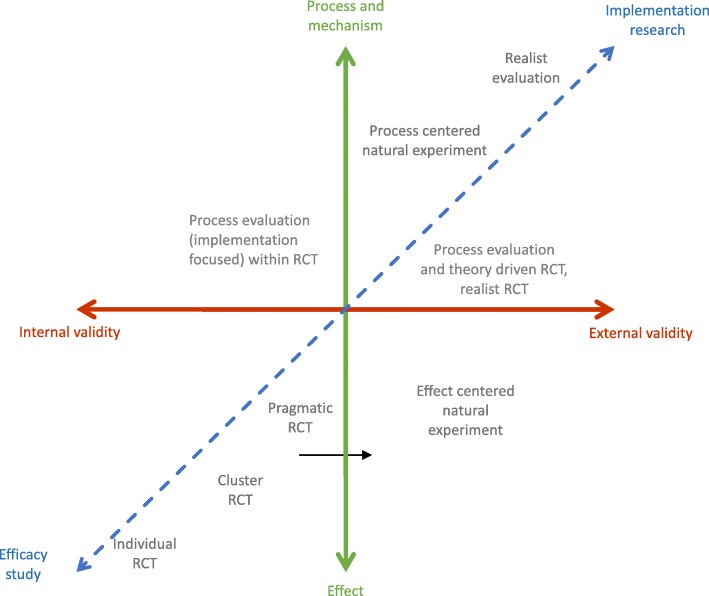
A framework situating designs according to evaluation questions. The x-axis presents the internal validity - external validity continuum. The continuum between process, mechanism and effects is represented on the y-axis. Finally, the transverse axis positions the various methods according to the research question, from efficacy studies to implementation research

All these methods present strengths and limitations that researchers have to consider when choosing the appropriate design in an evaluative context. The key issue for a researcher is to identify the most appropriate method. The complexity level may of course differ according to the domain and the object studied. More importantly, the research question must become the driving force for choosing the best evaluation design: if the researcher is interested in strictly demonstrating efficacy, then RCTs remain the best choice when it can be implemented; when external validity and an image of the real world are more important, other designs should be preferred. Similarly, the interest in the results, mechanisms and/or conditions of implementation [32] also guides the choice. We propose the Fig. 2 to situate each of the designs with respect to evaluation questions. The more close to the center, the more the design is approaching the point of balance between internal validity, external validity, effect evaluation and mechanism exploration. This framework do not have to be read as a fixed framework. Some study designs could fit into more than one quadrant of the figure. It allows to present design solely according to their specificities (internal/external validity, effect or mechanism evaluation). However, several designs may be combined to create a multidimensional evaluation. Thus a researcher may choose to use a pragmatic RCT and a process evaluation. Pragmatic RCT is robust to evaluate effectiveness of the intervention (its causal inference is high) and the process evaluation will allow him to inform about intervention mechanism and to produce generalizable results. In such case, pragmatic trials associated with process evaluation could be positioned closer to realist evaluation if the process evaluation is predominant in the evaluation.

## Conclusion

In conclusion, no “ideal” method can be proposed for evaluation of CIs, but a range of methods can be used in order to address various issues (evaluation of the effect of the intervention, examination of implementation, mechanisms of impact, and effects of contextual factors). They can therefore be used successively or combined at various steps of the evaluation approach, as evaluation is primarily a global approach comprising development of an intervention from a theoretical framework to various steps of evaluation, such as that proposed by the 2015 MRC guidance [25]. The growing interest of researchers in alternative methods and the development of their use must be accompanied by conceptual and methodological research studies in order to more clearly define their principles of use.

## Abbreviations

CI: Complex intervention
CRT: Cluster randomized trial
IRCT: Individual randomized controlled trials
MRC: Medical research council
PRISMA: Preferred Reporting Items for Systematic Reviews and Meta-Analyses
RCT: Randomized controlled trials
ToC: Theory of change
